# A Comprehensive Review on the Tribological Evaluation of Polyether Ether Ketone Pristine and Composite Coatings

**DOI:** 10.3390/polym16212994

**Published:** 2024-10-25

**Authors:** Amal A. Seenath, M. M. A. Baig, Jitendra Kumar Katiyar, Abdul Samad Mohammed

**Affiliations:** 1 Department of Mechanical Engineering, King Fahd University of Petroleum and Minerals, Dhahran 31261, Saudi Arabia; g202203660@kfupm.edu.sa (A.A.S.); mmurtuza@kfupm.edu.sa (M.M.A.B.); 2 Centre for Research Impact and Outcome, Chitkara University, Rajpura 140401, India; jitu1283@gmail.com; 3 Interdisciplinary Research Center-Advanced Materials, King Fahd University of Petroleum and Minerals, Dhahran 31261, Saudi Arabia

**Keywords:** PEEK, wear, friction, composites, coatings

## Abstract

Polymer coatings have gained a lot of attention in the recent past because of their ability to be easily coated on complex shapes, their low cost, and their ability to reduce friction as compared to other materials. Polyether ether ketone (PEEK) is one such high-performance polymer that has gained significant attention in recent years due to its exceptional mechanical properties, chemical resistance, and thermal stability making it a prominent candidate for applications in industries. However, PEEK in its pristine form exhibits poor wear resistance with a moderate coefficient of friction (0.30–0.38). Many attempts have been made by several researchers to improve its wear resistance and lower the COF by developing composite coatings. Hence, in this review, we aim to summarize and present in detail the tribological evaluation of pristine PEEK and PEEK composite coatings by discussing the various methods adopted by the researchers to improve the properties of PEEK, the different types of reinforcements and various dispersion techniques used to develop PEEK composite coatings. By consolidating and analyzing the existing body of knowledge, we also aim to offer valuable insights into the development of more durable, high-performance PEEK nanocomposite coatings for a broad range of tribological applications.

## 1. Introduction

**Figure 1:**
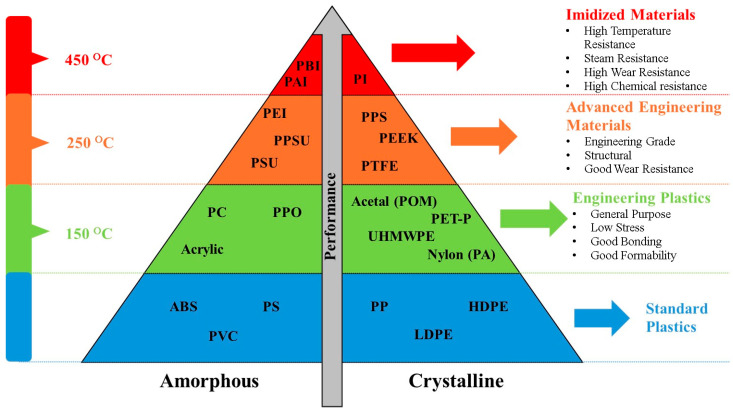
Polymer pyramid, listing PEEK with other thermoplastic polymers with regard to performance and formability.

Machines used in industries for various applications are often subjected to a harsh and challenging environment. Furthermore, the wearing out of machine components in relative motion, such as sliding, rolling or rubbing under different loading conditions, reduces their useful life. Polymeric coatings have been in the spotlight since the early 1930s and are used in several industrial applications to protect the contacting surfaces from wear and tear. Polymers are a special type of material and, due to their low cost, ease of manufacturing, and low coefficient of friction, were able to find their way into many industrial applications. Polyether ether ketone (PEEK) is one such semi-crystalline, high-performance thermoplastic engineering material that provides a distinctive combination of properties, including excellent thermal stability, good chemical resistance, exceptional mechanical strength, and reasonably good tribological properties [1,2,3,4]. This unique balance of properties makes PEEK exhibit excellent performance in harsh environments that other most commonly used polymers like polyamide (PA), polytetrafluoroethylene (PTFE), and ultra-high-molecular-weight polyethylene (UHMWPE) fail to withstand (Figure 1). Consequently, PEEK has become an exceptionally ideal choice for polymeric coatings for a wide variety of applications in the aerospace and automobile industries [5,6,7]. However, pristine PEEK exhibits a high coefficient of friction, between 0.30–0.38, in addition to a high wear rate [8]. Moreover, studies have shown that at elevated temperatures, PEEK tends to soften, which increases the wear rate, particularly when subjected to dynamic tribological loading [9]. In industrial applications where high temperatures and heavy loads are prevalent, such as in bearings or sliding components, PEEK’s performance as a tribological material can become compromised. To overcome this, several approaches have been adopted by researchers and one such approach was to tailor the properties of PEEK by incorporating it with different nanofillers [10,11]. Another approach was to reinforce PEEK with other polymers. Researchers have also conducted experiments by reinforcing PEEK with PTFE and UHMWPE [12,13,14] to reduce the coefficient of friction and obtain a higher wear resistance. Many attempts to produce hybrid composite polymer coatings [15,16,17,18] are also being made.

However, even with reinforcements, maintaining low friction and high wear resistance under such extreme conditions remains a challenge that continues to drive research in this area. Another significant issue in the development of PEEK composites was achieving uniform dispersion and strong adhesion of reinforcements within the PEEK matrix. Reinforcements like natural fibers [19], carbon nanotubes [20], or ceramic nanoparticles [21] are commonly added to improve the material’s wear resistance and reduce friction. Furthermore, environmental factors such as humidity, corrosion, and exposure to UV radiation also affect the long-term tribological performance of PEEK coatings [22,23,24]. As a result, the key aim of this review is to explore and evaluate the role of various reinforcements that have been incorporated into PEEK coatings to enhance their tribological performance. By reviewing studies that have employed different reinforcements, this paper will assess the effectiveness of these approaches in improving the performance of PEEK coatings, providing a detailed understanding of the relationship between reinforcement type, concentration, and resulting tribological properties. In addition to reinforcements, the coating procedure itself plays a significant role in determining the final performance of the developed coatings. Therefore, another important objective of this review is to examine the various coating techniques used in the literature. The third major area of focus in this review is the dispersion methodologies used to ensure that reinforcements are uniformly distributed within the PEEK matrix. Proper dispersion is essential for achieving consistent and reliable tribological performance, as poor dispersion can lead to the formation of agglomerates or inhomogeneities in the coating, which can significantly reduce wear resistance and lead to uneven frictional behavior. This review will assess the various dispersion techniques reported in the literature and report how these dispersion techniques have affected the overall properties of the coatings.

To the best of our knowledge, no previous review has focused exclusively on PEEK coatings, specifically addressing the effect of reinforcements, coating procedures, and dispersion methodologies on their tribological properties. This highlights a gap in the existing literature, which this review aims to fill by providing a comprehensive analysis of these aspects. However, a few review papers summarizing the tribological behavior of PEEK bulk composites [12,25,26,27,28,29] have been reported. However, it is to be noted that the properties of the bulk differ significantly from the coatings in the tribological evaluation as the tribological assessment of coatings depends upon the substrate preparation and the adhesion factor, which rely on methods including cleanliness and pre/post-heat treatments involved etc. Hence, the present review aims to focus on summarizing the effect of various factors on the tribological performance of PEEK coatings, either in the pristine form or composite form.

**Figure 2:**
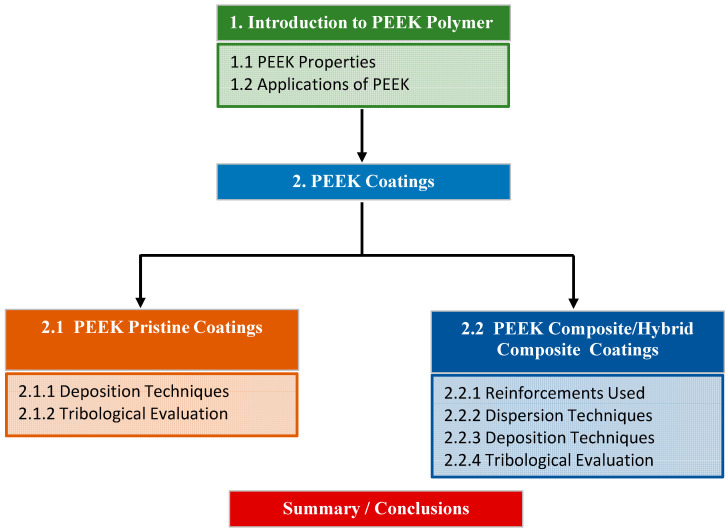
Bird’s view of the arrangement of the review.

Figure 2 intends to provide the readers with a bird’s view of the different sections in the paper for easy understanding.

### 1.1. PEEK Properties

PEEK is a high-impact polymer showcasing exceptional mechanical and physical properties. PEEK polymers are manufactured by step-growth polymerization by the dialkylation of bisphenol salts. PEEK has low surface energy, due to which it displays a low adsorption rate. PEEK was found to have a low water solubility range of 0.5 wt%, even at temperatures around 260 °C [30]. PEEK, a semi-crystalline material, exhibits an elastic modulus of around 3.1 GPa [21]. Despite its excellent mechanical and physical properties, PEEK exhibits poor friction and wear performance. Table 1 provides in-depth information on the various properties PEEK exhibits as well as on its chemical structure. Figure 1 encapsulates the advantages and limitations of using PEEK as a tribo-material as a bulk and protective coating. It also suggests methods to be considered in successfully producing PEEK as a tribologist’s polymer.

### 1.2. Applications of PEEK

Driven by the fuel efficiency demand, there is much impetus to replace metallic parts with lighter alternatives, such as plastic or polymer materials. As discussed, the exceptional behavior of PEEK in extreme conditions makes it a suitable replacement in the manufacturing of automobile parts like seals, washers, and bearings. Aluminum is a popular choice in the aerospace industry due to its lightweight. PEEK, being a lighter material than aluminum, has become an alternative for the latter, especially in the aerospace industry [7]. Currently, PEEK has also found its way into medical applications due to its biocompatible nature, which stems from its insolubility in most polymeric solvents [26,31,32,33]. It is also an appropriate choice for dental implants in composite form [17]. Being an excellent insulator, it has found its way into electrical instruments operating at high temperatures, such as handles of soldering irons [34]. Due to its exceptional chemical and heat resistance, it has also been used extensively in the oil and gas applications [6]. Table 2 provides an in-depth overview of its employment in various spans of applications varying from small to large-scale industries. In order to obtain a clearer understanding of the industrial landscape surrounding PEEK, a detailed overview of key manufacturers of PEEK is also provided in Table 3.

**Figure 3:**
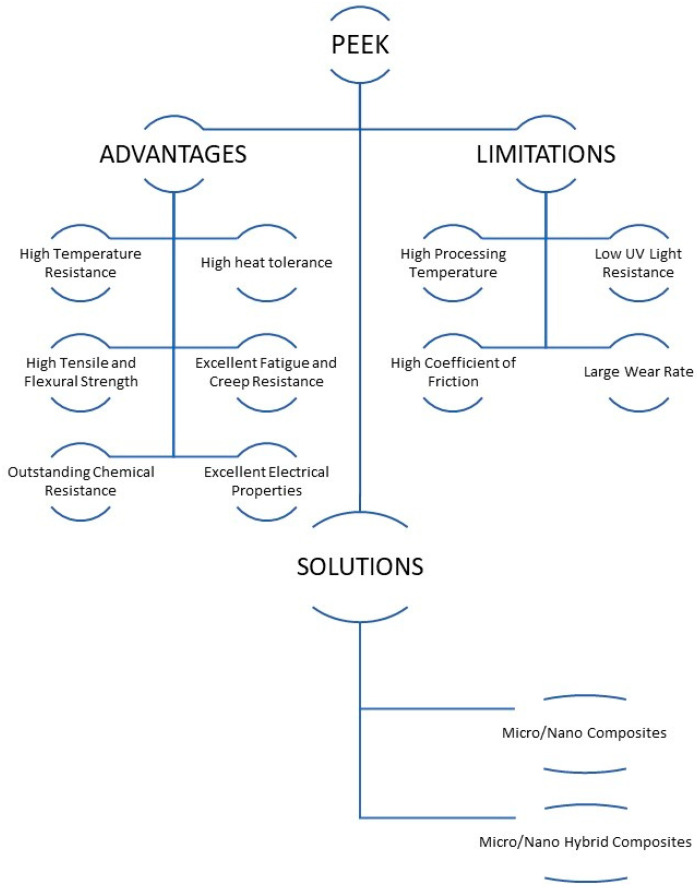
Advantages/limitations of PEEK and the possible solutions to overcome these challenges.

Figure 3 summarizes the advantages and limitations of PEEK as a polymer. As can be seen, PEEK has a very high-temperature resistance compared to other polymers, which makes it attractive for high-temperature applications. In addition to its high-temperature resistance, it also has a few attractive mechanical properties, such as high tensile/flexural strength and excellent fatigue and creep resistance, which are required in mechanical/tribological applications [35]. All the above properties, coupled with its good chemical resistance and good electrical properties, make PEEK very attractive for different industrial applications. However, it is to be noted that it does suffer from a few limitations, such as, it shows a higher coefficient of friction and higher wear rates as compared to a few polymers such as polytertrafluoro ethylene (PTFE) and ultra-high molecular weight polyethylene (UHMWPE). However, these limitations can be overcome by fabricating PEEK composites by reinforcing PEEK with a suitable filler, which helps in improving its tribological properties, such as lower coefficient of friction and higher wear resistance.

## 2. PEEK Coatings

Currently, many researchers are focusing on improving the characteristics of PEEK to be used as a bulk material as well as thin coatings on numerous substrates. Since our area of interest is purely on PEEK coatings, an in-depth analysis of the various studies performed by the researchers on producing these coatings to obtain optimum tribological performance is presented.

### 2.1. Pristine PEEK Coatings

Limited works have been carried out by using pristine PEEK as a coating on different substrates, especially due to their high coefficient of friction and high wear. The following sections present an in-depth analysis of various deposition techniques and their relative merits.

#### 2.1.1. Deposition Techniques of Pristine PEEK Coatings

The selection of an appropriate deposition technique, which depends on respective material properties, is critical to coating performance. Flame-spray [1,30,36] and electrophoretic [37] techniques reported in literature for depositing pristine PEEK on metallic substrates are presented in Table 4. It was noted that mere deposition results in poor mechanical properties due to the formation of highly porous films with weak adhesion to the substrate. Post-deposition treatment processes such as laser remelting and heat treatment in an oven emerged to be effective in eliminating/diminishing pores, rendering dense, homogeneous, and continuous coatings. However, process parameters must be optimized to obtain desired coating characteristics. Moreover, the type of metallic substrate was also reported to affect coating characteristics, especially in the case of laser remelting processes.

#### 2.1.2. Tribological Evaluation of Pristine PEEK Coatings

The tribological evaluation mainly includes friction, wear, and scratch tests.

Zhang et al. [30] conducted the friction test on CO_2_ and Nd: YAG remelted flame-sprayed PEEK coating by using an oscillating ball-on-disc tribometer. The CO_2_ laser remelting resulted in a semi-crystalline structure, whereas Nd: YAG laser remelting resulted in the amorphous structure of the coating. The tests were performed under the applied load of 2 N and speed of 0.3 m/s against a steel ball in dry sliding conditions. It was also observed that the peak value of the coefficient of friction obtained with both the CO_2_ and Nd: YAG laser-remelted coatings was about 0.35. However, the CO_2_ laser remelted coating exhibited a slightly lower friction coefficient. This was ascribed to the difference in the crystal structure of remelted coatings. It is to be noted that the reported value of friction coefficient (~0.35) is higher compared to other polymer coatings.

Similarly, Kruk et al. [37] studied the effect of PEEK coating in improving the tribological performance of the Ti-6Al-4V substrate. A general improvement in wear resistance of more than 100 times and a reduction in friction coefficient of 2 times (from 0.72 to 0.27) was reported with PEEK coating on the substrate. Furthermore, the effect of coating structure on room-temperature mechanical and scratch properties along with tribological properties (at room temperature, 150 °C and 260 °C) was also studied. Furnace cooling of the coated sample after the post-deposition heat treatment resulted in a semi-crystalline structure, whereas water cooling resulted in an amorphous structure of the coating. Coatings with semi-crystalline structure exhibited greater micro-hardness, elastic modulus and scratch resistance in comparison to amorphous structure. Additionally, higher wear resistance and relatively lower friction coefficient were also reported at all the test temperatures. Therefore, slow cooling rates after post-deposition heat treatment appear to have a positive effect on tribological properties.

The improvement in properties of base material with PEEK coatings indicated in the above studies remains far inferior in comparison to other polymeric substances like UHMWPE and PTFE. A high value of the coefficient of friction and wear rate were observed in comparison to other polymeric coatings. Therefore, the necessary reduction in friction and wear is achieved by adding fillers onto the PEEK feedstock to develop a composite/hybrid coating technology. A detailed discussion of the reinforcements used, the various dispersion and deposition techniques practiced, and the tribological evaluations are presented in the following sections. These are the contributing parameters in obtaining the desired characteristics of the composite/hybrid PEEK coatings so that they can be successfully used in a variety of applications.

### 2.2. Composite/Hybrid Composite PEEK Coatings

Modern life challenges of global warming, a sustainable environment, energy consumption, etc. have inspired the researchers to explore possible means to reduce friction and wear between various sliding components. The lubrication strategies have been the most common focus of research interest. Currently used techniques involve the usage of hard coatings and lubricants with additives to reduce wear and friction. However, these techniques suffer from their own drawbacks. The lubricant additives contain sulphur and phosphorous compounds which are toxic in nature and have serious effects on the environment and health. The hard coatings suffer from a number of limitations like poor adhesion with the substrate, high deposition costs, complex deposition techniques, etc. [38].

To overcome these challenges, numerous kinds of research are conducted on thin polymer coatings. Polymer coatings are characterized by low friction, wear, low deposition cost, simple deposition techniques, etc. As a result, polymers like PTFE, UHMWPE, PEEK, etc. are being widely investigated. Among these, PEEK stands out, showing excellent thermal and mechanical properties, which allows it to be widely used in extreme environmental conditions. However, as discussed earlier, pristine PEEK as thin coatings tend to show a high coefficient of friction and high wear rate. In order to enhance the properties, fillers are added to the PEEK feedstock as reinforcements. Research has been conducted on incorporating PTFE, CF, graphite, CNT, etc. with PEEK so as to take advantage of the individual properties of each filler into one product. The quality and enhancement in the performance of the hybrid/composite coatings depend on the shape and size of the reinforcements used, the dispersion technique as well as the deposition methods employed.

#### 2.2.1. Various Reinforcements Used to Fabricate the Composite/Hybrid Composite PEEK Coatings

The reinforcements that have been used to enhance the properties of PEEK coatings can be classified under seven groups: bioactive, ceramic, carbon based, refractory, metallic, polymeric and mineral based materials as shown in Table 5. It also shows the characteristics of respective reinforcements that can impart the desired changes in properties of PEEK coatings. Additionally, certain reinforcements that have been classified under ceramic, refractory, and metallic materials are reported by several studies to also exhibit bio-activity, biocompatibility, cytocompatibility, hemocompatibility, anti-bacterial, anti-microbial, and anti-inflammatory effects.

The selection of fillers or reinforcements depends upon the targeted applications. Cao et al. [62] reinforced PEEK with tantalum nanoparticles to fabricate a composite coating to be used in bone repair applications. PEEK, known for its high load-bearing capability, was found to provide poor strength for artificial knee joints. On the other hand, tantalum is a biocompatible metal with excellent strength and corrosion resistance [65,67,68]. Therefore, mixing tantalum with PEEK enhanced the high-pressure performance as a load-bearing substitute for bone. Similarly, Zhu et al. [69] added PTFE nanofillers to PEEK in order to improve the mechanical strength and enhance the hydrophobic nature. Though PTFE shows extremely low values of coefficient of friction, it demonstrates a low melting point. On the other hand, PEEK has exceptional thermal properties in comparison to other polymers. Therefore, PEEK when reinforced with PTFE was found not only to improve friction performance but also to show good thermal properties for the composite film prepared. Incorporating alumina was found to produce wear-resistant coatings [70,71] and increase the pitting potential of the substrates. Similarly, many researchers have conducted numerous experimental analyses with different types of fillers including SiO_2_ [51], SiC [53], TiO_2_ [72], PTFE/Graphite [15], etc., and observed that the desired changes in properties can be achieved by optimizing various parameters including the deposition techniques, filler materials, parameters selected, etc.

#### 2.2.2. Dispersion Techniques of the Reinforcements Within the PEEK Matrix

The reinforcements selected must be thoroughly and uniformly distributed within the PEEK matrix in order to obtain the targeted result. Non-uniform distribution can lead to the agglomeration of the particles, which will result in degrading the properties including the adhesion between film and substrate. Hence, a variety of dispersion techniques have been used to disperse the reinforcements within the PEEK matrix for uniform dispersion, which are listed and discussed below.

a. **Wet Mixing**

Ethanol is used as a solvent for dispersion of powder particles. After adding the powder particles to ethanol, the mixture is sonicated in either an ultrasonic bath or by using a probe-sonicator. The sonication is performed to deagglomerate and uniformly disperse the powder particles. It is followed by mixing with magnetic stirring of the suspension, as shown in Table 6. In some studies, mixing by magnetic stirring is performed first which is followed by sonication [33,39,40,44,45]. The prepared suspension is directly used in the electrophoretic coating process. On the other hand, for electrostatic and flame-spray coating processes which require feed stock in the form of dry powder, the suspension is dried in an oven to completely evaporate the solvent. In preparation of composite powder for electrostatic spray coating [73], after adding the filler to ethanol, the solution was sonicated using a probe sonicator for 30 min. After the deagglomeration and uniform dispersion of filler, the matrix powder (PEEK) was added followed by sonication for another 30 min. Similarly, in the preparation of hybrid powder for electrostatic coating, the solution containing primary composite powders were magnetically stirred for 10 min. Then the secondary filler was added to this solution followed by magnetic stirring for 15 min. The different sonication and magnetic stirring times presented in Table 6 indicates that the choice of time is based on the researcher’s practical observation.

b. **Dry Mixing**

Dry mixing appears to be the preferred dispersion method when the coating process requires feed stock to be in dry powder form as in the case of electrostatic and flame-spray coating processes. It is generally accomplished using ball mill. Zhu et al. [69] adopted the mechanical dispersion technique of blending. This mixture was then blended on a high-speed blender at room temperature. Prior to spraying the composite mixture, it was dried in a vacuum oven at 150 °C for 4h so as to remove any moisture content. Hedayati et al. [51] used Ball milling for uniform blending of SiO_2_ particles with PEEK powder. The powders were physically blended and milled at a planetary ball milling machine for 15 h at 280 rpm. Similarly, Li et al. [20] also blended CNT particles with PEEK powders at a speed of 150 rpm for 90 min.

c. **Effect of Dispersion Technique on Coating Morphology**

Li et al. [20] prepared CNTs/PEEK nanocomposite coatings using the flame-spray technique. In order to study the effect of dispersion technique on coating morphology, the feed stock of CNTs/PEEK powders were mixed using mechanical blending (MB) and ultrasonic dispersion (UD) method. The coatings formed using MB powders showed several micro-voids which increased in size and number with increasing CNTs content. On the contrary, coatings formed using UD powders showed very few voids. Moreover, the coatings formed using MB powders showed larger agglomeration of CNTs in comparison to the coating prepared using UD powders. This larger agglomeration of CNTs in coating prepared using MB powders was responsible for the formation of large voids in the coating.

#### 2.2.3. Deposition Techniques Used for Composite/Hybrid Composite PEEK Coatings

The deposition is by far the most important procedure concerning the tribologist for coating a substrate. Various types of coating techniques are now practiced by many researchers on depositing PEEK composites over the substrate. However, prior to the deposition technique, one must be very careful in cleaning the substrate on which the coating has to be deposited. This is mainly done so as to make the surface free from any foreign contaminants, impurities, oil, etc., and to improve the adhesion between the sample and coating. Cleaning the sample with acetone and drying it under dry air is the most common technique practiced in all our literature studies. After cleaning the substrate, another major step is the pre-treatment of the substrate. This is mainly done to create the sample surface ready according to the researcher’s requirement for particular applications. Some surfaces are made rough, particularly so that there will be a higher degree of adhesion of between the coating and the sample. The degree of surface roughness required varies from material to material and application needs. Grit blasting is one of the most common pre-treatment techniques used in the deposition of PEEK and its composite coatings. Grit blasting helps in cleaning the substrate and attaining the required surface roughness for the researcher’s necessity. It can be carried out with a number of abrasives, like Al_2_O_3_, SiC, etc., depending on the surface roughness needed for the experimental investigation.

The deposition procedure follows after the cleaning and pretreatment of the substrate. The post-heat treatment must be performed after deposition to eliminate porosity and further densify the deposit. Over the years, researchers have conducted numerous investigations on PEEK composite coatings deposited by various techniques, which are discussed below.

d. **Electrophoretic technique**

**Figure 4:**
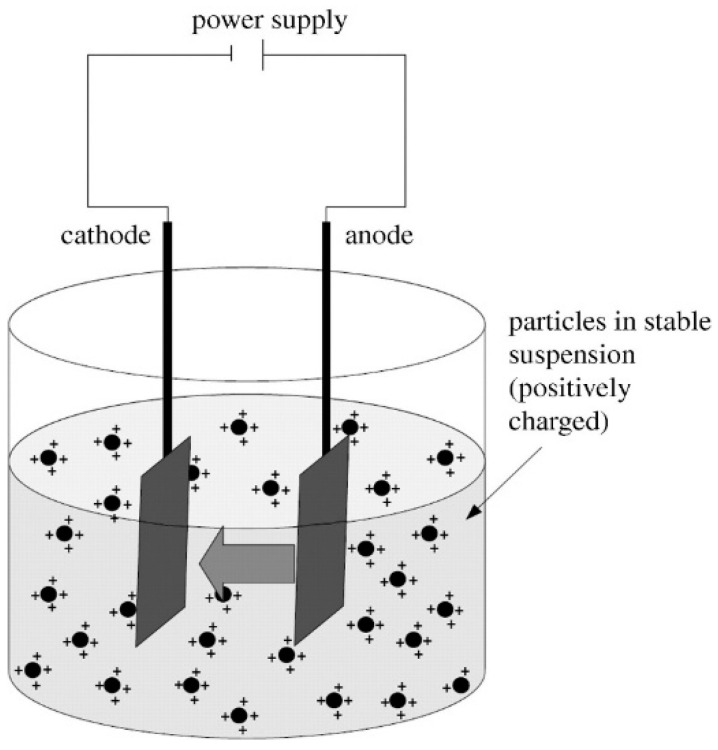
Illustration of electrophoretic deposition technique [33].

Electrophoretic deposition has been a common technique used to deposit PEEK nanocomposite on titanium and stainless steel substrates [15,33,39,40,41,42,44,45,46,47,48,49,50,51,52,53,54,55,56,57,58,59,60,61,62,63,64,65,66,72,75]. It is a material deposition technique that can be performed only on conductive materials using the principle of electrophoresis. The creation of the suspension containing the hybrid/composite particles in non-aqueous liquid solvent preferably ethanol is the first step in the process [75]. Some researchers have also reported using co-solvent, i.e., 95 vol% ethanol and 5 vol% isopropanol [59,60]. Chitosan was commonly added as a dispersant/surfactant or charging agent to the suspension medium to promote uniform dispersion and avoid agglomeration of particles. Hydrochloric acid [39,40,72], citric acid [42,49,54,57,58], and sodium hydroxide [39,40,49,54,57,58,72] were used as stabilizers to balance the pH of the suspension. The conductive substrate is then immersed in the suspension as cathode. A counter electrode made of inert material used as anode is also immersed in the suspension. The charged particles in the suspension are attracted and deposited as a coating on oppositely charged cathode on the application of a DC electric field [76] as illustrated in Figure 4. It is to be noted that the water is not used as a suspension medium as the application of electric field results in the evolution of hydrogen and oxygen gases at the electrodes which adversely effects the quality of the coating [48]. This technique has gained more popularity in the recent past as the deposition can be made on any shape of the substrate with a little modification of the electrode. Following this, many researchers have adopted this technique for depositing PEEK composite coatings intended for bio-medical applications due to the non-uniform shape and the limited size of the implant.

**Figure 5:**
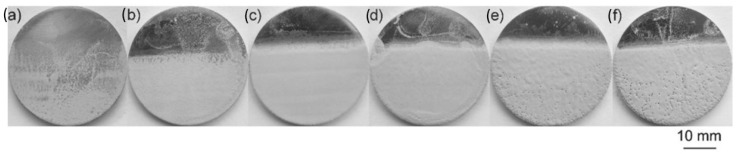
Macroscopic images HA/MoS_2_/PEEK hybrid coatings deposited on Ti-13Nb-13Zr alloy substrates at applied voltage of (**a**) 50 V, (**b**) 70 V, (**c**) 90 V, (**d**) 110 V, (**e**) 130 V and (**f**) 150 V at a constant deposition time of 30 s [40].

As is evident from Table 7, many researchers have shown that adjusting the processing parameters such as applied voltage or deposition time or both can easily control the characteristics of deposited coating. For example, HA1/MoS_2_/PEEK hybrid coatings were deposited on Ti-13Nb-13Zr alloy substrates in a voltage range of 50 V to 150 V at a constant deposition time of 30 s as shown in Figure 5. Thin and inhomogeneous coatings were deposited at applied voltages of 50 V and 70 V whereas dense and homogeneous coatings were at voltages of 90 V and 110 V. Further increases in applied voltages to 130 V and 150 V resulted in increases in the pore density and size.

**Figure 6:**
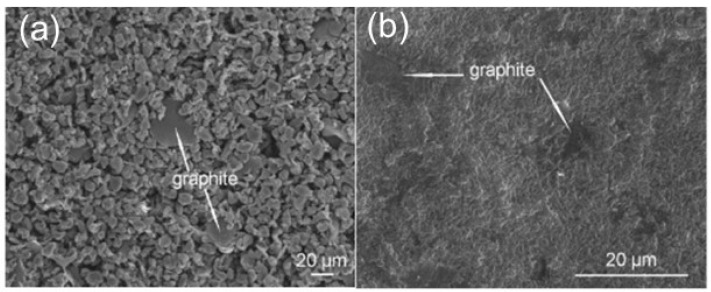
SEM images of the graphite/PEEK (**a**) as deposited and (**b**) and heat-treated coatings [58].

The deposited coatings are subjected to heat treatment after air dying for rendering it dense and homogeneous and to improve its adhesion to the substrate. The figure shows the graphite/PEEK composite coatings immediately after deposition and after subjecting it to the post heat treatment [58]. Figure 6a shows that the substrate is uniformly covered by the PEEK particles with sporadic distribution of graphite particles immediately after deposition. PEEK changes its morphology upon post heat treatment to continuous coating matrix with graphite particles embedded as shown in Figure 5b. Moreover, the coating became dense with no cracks or pores after heat treatment. Finally, the coatings are furnace cooled at a steady rate naturally at room temperature or when quenched in water to room temperature. The cooling rate affects the degree of crystallinity of coating which may have different effects on mechanical and tribological properties.

Table 7 shows that in order to obtain thick and uniform coatings, several researchers picked optimal processing parameters after depositing coatings at varying applied voltages for varying deposition time. Moreover, Table 6 shows a huge variation in the optimal applied voltages and the deposition times reported. This indicates that the optimal processing parameters are unique to the respective coating setup, suspension medium and coating composition. It is observed that the post heat treatment was performed at a temperature above the melting point and below the degradation temperature of PEEK.

e. **Electrostatic spray coating**

**Figure 7:**
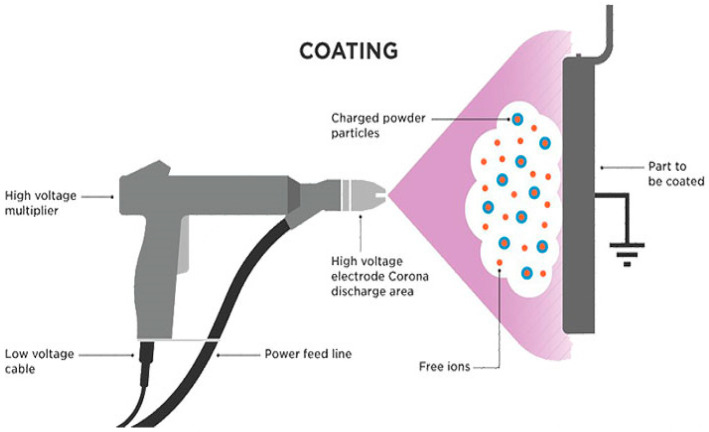
SIllustration of electrostatic spray coating [79].

Electrostatic spray coating is a deposition technique that utilizes the principles of electrostatics to apply coatings onto various substrates (Figure 7). Using an electrostatic spray gun, the desired powder is charged with an electrostatic charge during this procedure. The polarity of the electrostatic field determines whether the powder particles pick up a positive or negative charge [77]. As the charged powder particles are propelled toward the substrate, they are attracted to the grounded surface, resulting in a uniform and controlled deposition of the coating material [78], as illustrated in Figure 7. This is a very simple and cost-effective coating technique used to coat conductive materials, like titanium alloys [17], plain carbon steel [51], and stainless steel [69,72,73], with PEEK composite coatings intended mostly for industrial tribological applications. By optimizing a number of variables, including the powder flow rate, electrostatic voltage, spray distance, and deposition time, it is possible to obtain the desired coating thickness and uniformity. The optimum spraying parameters reported in the literature are presented in Table 8. Moreover, to improve the coating’s qualities further, the coated substrate must be subjected to a post-processing procedure like heat treatment once the coating process is finished. Additionally, heat treatment can be used to improve the coating’s structure and crystallinity, which will improve its performance in particular applications.

f. **Flame-Spray Coating Technique:**

**Figure 8:**
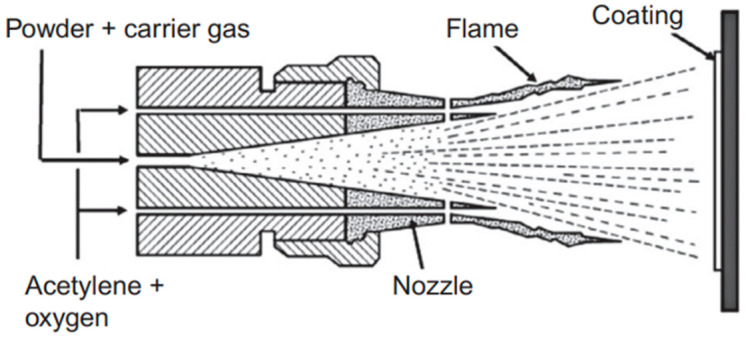
Illustration of flame-spraying technique [82].

Another widely used dispersion technique used to deposit coatings on the desired substrate is the flame-spray coating technique. In this procedure (see Figure 8), a fuel gas such as propane or acetylene is combined with oxygen to create a high-temperature flame. The heat source for melting the powder particles is this flame. In addition, the powder particles are propelled into the flame and towards the substrate by a carrier gas, usually nitrogen [80]. The powder particles melt and atomize when they come into contact with the flame. Melting powder particles produce molten droplets due to the extreme heat and these droplets are simultaneously broken up into smaller particles by the force of the carrier gas, resulting in a fine and homogeneous material dispersion [81]. They quickly cool and solidify upon coming into contact with the substrate surface, creating a thin coating layer. Throughout this process, the temperature of the substrate is closely monitored to maximize adhesion and reduce thermal stress, guaranteeing the longevity and integrity of the coating. The number of passes needed in the flame-spray coating process depends on the characteristics and thickness of the desired coating. After the substrate has been coated to the appropriate thickness, it is allowed to cool to ambient temperature.

The flame-spraying technique was also used by the researchers to deposit PEEK nanocomposite powder over the steel substrates [46,55,61]. It is also a very simple and cost-effective technique that was used to deposit PEEK nanocomposite coatings intended for industrial tribological applications. The spraying parameters reported in various studies are presented in Table 9. It is noted that this process does not require post heating but requires preheating. It’s a one-step process where the pre-heat is also done by the flame itself. Therefore, this technique is more appropriate for coating substrates too large to be heated in oven.

#### 2.2.4. Tribological Evaluation of the Composite/Hybrid Composite PEEK Coatings

PEEK based composite/hybrid coatings on metallic substrates reported in literature can be classified as coatings for biostructural, bio-tribological, and tribological applications. The coatings designed for biostructural applications (structural component of implant) focus on enhancement of bioactivity, antibacterial properties, corrosion resistance, and adhesive strength. The coatings designed for bio-tribological applications where the implant is expected to experience relative motion in service contain hard particles which are biocompatible. They focus on enhancement of friction and wear properties along with electro-mechanical properties. The coatings designed for tribological applications in industry focus on improvement in corrosion, friction and wear properties. All these three categories will be reviewed in terms of tribological performance in this section.

A. **Bio structural Applications**

The coatings designed for bio structural applications (structural component of implant) focus on enhancement of bioactivity, antibacterial properties, corrosion resistance and adhesive strength. Since the focus of this review is tribological evaluation, only those studies that reported properties relevant to tribological performance like hardness and scratch resistance were presented.

Kusmierczyk et al. [39] deposited sulfonated PEEK/hydroxyapatite(n-HA)/zinc sulfide (ZnS) hybrid composite coating over Zr-2.5Nb zirconium alloy substrate using an electrophoretic deposition technique. Heat treatment of the dried coatings was carried out at 450 °C for 30 min at a heating rate of 15 °C/min followed by cooling it at a constant rate of 2 °C/min. The heat treatment at a temperature of 450 °C resulted in the sulfonation process thereby leading to the formation of amorphous PEEK. The micro-scratch tests were conducted using a Rockwell C diamond stylus with an apex angle of 120° and a tip radius of 200 µm. The test specimens were linearly loaded from 0.01 N to 30 N with a speed of 5 mm/min followed by a scratch length of 5 mm. The scratch tests and the hardness tests concluded that the addition of HA and ZnS demonstrated excellent adhesion and scratch resistance when compared to PEEK coatings. Moreover, the addition of HA has increased the hardness and improved the elasticity of the coatings.

Kusmierczyk et al. [40] deposited PEEK 704/hydroxyapatite (HA)/molybdenum disulfide (MoS_2_) hybrid nanocomposite coating over Ti-13Nb-13Zr alloy substrate using an electrophoretic deposition technique. Heat treatment of the dried coatings was carried out at 390 °C for 40 min at a heating rate of 4.5 °C/min followed by cooling it at a constant rate of 2 °C/min. The micro-scratch tests were conducted using a Rockwell C diamond stylus with a tip radius of 200 µm. The test specimens were linearly loaded from 0.01 N to 15 N with a constant velocity of 5 mm/min on a scratch length of 5 mm. Investigations were conducted using two different particle sizes of HA, namely with HA1 having a mean particle size of 43 nm and HA2 having a mean particle size of 200 nm. Scratch testing’s have concluded that there were no noticeable effects of the particle size on the tribological properties of the coatings. Both exhibited similar resistance to scratch despite the small thickness of the coatings (~30 µm–35 µm). However, the coatings with smaller particle sizes of HA (HA1) exhibited a slightly higher hardness value (0.32 GPa) than the coatings with larger particle sizes of HA (HA2) (0.30 GPa).

Garrido et al. [43] deposited PEEK/bioglass composite coating over PEEK substrates using a cold gas spray (CGS) technique. The concentration of bioglass in coating was between 10 to 50 wt%. In this study, two different coating thicknesses were studied by varying the traverse gun speed. A thick coating (~700–900 µm) was obtained by using a low traverse gun speed (80 mm/s) and a thin coating (~200–300 µm) was obtained by a high traverse speed (240 mm/s). The tribological testing on the thick coatings (80 mm/s) were conducted on a ball-on-disk arrangement at a load of 5 N and a constant velocity of 133 rpm for a total sliding distance of 1000 m. Alumina balls with a diameter of 9 mm were used. The presence of glass in the coatings has increased the wear resistance thereby exhibiting a slight decrease in the COF (from 0.51 to 0.50). A reduction in the volume of lost material (more than 70% when compared to the pure PEEK coatings) was also observed. The coating containing 50 vol.% of bioglass showed the highest wear resistance and hardness, which resulted in the conclusion that the glass particles prevented the polymeric particles from detaching.

Corni et al. [46] deposited Al_2_O_3_/PEEK nanocomposite on 316 stainless steel substrate using electrophoretic technique. The concentration of Al_2_O_3_ in coating was between 20 to 70 wt%. The results of scratch tests indicate that the filler concentration was too high to achieve improvement in scratch resistance. It was suggested to reduce filler concentration to improve mechanical properties. The lower filler concentration ensures greater PEEK–substrate contact area and PEEK–Al_2_O_3_ interface with no/negligible Al_2_O_3_-Al_2_O_3_ particle contact.

Fiolek et al. [58] deposited PEEK/graphite composite coating over Ti-6Al-4V substrate using an electrophoretic deposition technique. The heat treatment consisted of heating the coated samples with the furnace to a temperature of 390 °C at a constant rate of 4.5 °C/min for 40 min and then cooling with the furnace to room temperature with a cooling rate of 2 °C/min. The scratch tests were conducted using a Rockwell C diamond stylus with a tip radius of 200 µm. The test specimens were linearly loaded from 0.01 N to 30 N with a constant velocity of 5 mm/min on a scratch length of 5 mm. The introduction of graphite fillers was found to reduce the scratch resistance property of the coatings when compared to the unfilled PEEK coatings. It was also observed that the penetration depth of the indenter at the maximum load (30 N) was 75 µm and 65 µm for the composite and pure PEEK coatings, respectively. The introduction of graphite fillers has however resulted in the reduction of COF. A low COF of 0.10 was observed for the PEEK/graphite composite coating in comparison to the COF of 0.12 for pure PEEK coating. The parallel orientation of the graphite particles embedded in the polymer were found to be the most advantageous arrangement in terms of improving the friction process.

Fiolek et al. [66] deposited PTFE/PEEK composite coating on Ti-6Al-4V substrates using an electrophoretic technique. After drying, the coating was heat-treated to a temperature of 450 °C for 20 min, followed by furnace cooling at a rate of 2 °C/min. The friction and wear tests were performed against Al_2_O_3_ ball using a ball on disk tribometer. The tests were performed under a normal load of 5 N and a sliding velocity of 0.05 m/s at room temperature, 150 °C, and 260 °C for a sliding distance of 2000 m. A low coefficient of friction of 0.1 was recorded for PTFE/PEEK composite coatings in comparison to coefficients of friction of 0.27 and 0.33 for pure semi-crystalline PEEK and pure amorphous PEEK coatings respectively for room-temperature testing. It also showed a seven-times-lower wear rate than that of pure semi-crystalline PEEK coatings at room temperature. For tests performed at 150 °C, the coefficient of friction increased to 0.17 and the wear rate increased by four times than that at room temperature for the PTFE/PEEK composite coating. The formation of self-lubricating polymer film due to the presence of PTFE resulted in a low coefficient of friction and wear rate. The coating could not withstand the test at 260 °C.

B. **Bio Tribological Applications**

Bio tribology investigates the friction, wear, and lubrication phenomena occurring within biological systems and at the interfaces between biological tissues and synthetic materials. Biotribology testing includes a range of techniques including wear testing, friction testing, biological testing, surface characterization, and lubrication studies. These tests aim to evaluate the performance, durability, and biocompatibility of materials and devices, providing insights crucial for the design and development of safer and more effective biomedical solutions.

Qin et al. [17] deposited graphene oxide (GO)/carbon fibers (CF)/PEEK hybrid composite coatings on Ti-6Al-4V substrate by electrostatic powder spray technique. After spraying, the samples were put into a furnace at 385 °C for post-heat treatment for 60 min at a constant heating rate of 5 °C/min followed by cooling naturally to room temperature. Friction tests were conducted using a vertical reciprocating wear testing machine with Si_3_N_4_ as the counter-face ball (Ø = 5 mm) at a normal load of 5 N and a sliding speed of 2 Hz for 60 min. A low coefficient of friction of 0.08 was recorded for GO/CF/PEEK hybrid composite coatings in comparison to coefficients of friction of 0.19 for pure PEEK coatings. It was also observed that the addition of nanofillers to the PEEK coatings improved the hardness values (from 17.17 HV to 30.53 HV) in addition to the decreased COF and enhanced wear resistance.

Rehman et al. [33] deposited PEEK/bioactive glass composite coating over 316 L stainless steel substrates using an electrophoretic deposition technique. After the coating, the samples were sintered at temperatures of 355 °C, 375 °C, 400 °C, and 450 °C for 30 min at a heating rate of 2 °C/min followed by cooling to ambient temperature at a cooling rate of 20 °C/min. Wear tests were performed using a pin-on-disc method at a normal load of 7 N. The scratch tests concluded that the coating heat treated at 400 °C was found to exhibit the highest wear resistance at the implantation load. However, the addition of bioactive glass has increased the COF when compared to pure PEEK coating (from 0.26 to 0.37). Howervr, this is in direct relation to the wear behavior of the coating for designing the implant in the study performed.

Moskalewicz et al. [49] deposited Al_2_O_3_/PEEK nanocomposite on Ti-13Nb-13Zr substrate using an electrophoretic deposition technique. The coated samples were dried at room temperature after EPD and then heated in a furnace for 20 min at a temperature of 350 °C (heating rate of 4.5 °C/min and cooling rate of 2.7 °C/min). A ball-on-disk setup was used to study the friction and wear properties of the coatings with alumina (Al_2_O_3_) balls of 6 mm diameter as the counter face. Testing was performed at a normal load of 5 N at 120 rpm rotation speed, corresponding to a sliding distance of 1000 m. A low coefficient of friction of 0.29 was recorded for Al_2_O_3_/PEEK composite coating in comparison to coefficients of friction of 0.37 for pure PEEK coating. It also showed a two-times-lower specific wear rate than that of pure PEEK coating. The mean hardness and elastic modulus of the Al_2_O_3_/PEEK coatings were equal to 0.34 ± 0.03 GPa and 6.2 ± 0.3 GPa, respectively. These values were higher by 15% to 25% compared with the PEEK coating. The addition of ringer solution (a common electrolyte for corrosion study) was found to reduce the COF to a much lower value under lubricated testing conditions.

Moskalewicz et al. [54] deposited Si_3_N_4_/PEEK composite coatings on Ti-13Nb-13Zr substrate by electrophoretic technique. Two different dispersants, type 1 being polyethyleneimine (PEI) and type 2 being chitosan (CHIT), were used to make the powder, after deposition, the coatings were dried and then heat treated at a temperature of 355 °C for 30 min (heating rate of the samples 4.5 °C/min) and were cooled down with the furnace (cooling rate of 2 °C/min). The scratch tests were conducted using a Rockwell C diamond stylus with a tip radius of 200 µm. The test specimens were linearly loaded from 0.01 N to 30 N with a scratch speed of 5 mm/min on a scratch length of 5 mm. The scratch tests showed that the Si_3_N_4_/PEEK coating deposited from the suspension containing PEI exhibited poor and much lower scratch resistance than the Si_3_N_4_/PEEK coating deposited from the suspension stabilized by the CHIT polyelectrolyte, thereby concluding that CHIT was found to be an effective surfactant to disperse the Si_3_N_4_ nanoparticles and PEEK microparticles in the EPD process.

Lio et al. [15] deposited PTFE/PEEK/graphite hybrid nanocomposite coating on titanium substrates using an electrophoretic technique. After drying the coating at room temperature for 12 h, it was heat-treated to a temperature of 390 °C for 60 min followed by cooling to ambient temperature. The friction and wear tests were performed against using a ball on disk tribometer. The tests were performed under a normal load of 5 N and 10 N at a sliding velocity of 0.05 m/s at for a sliding distance of 1080 m. A low coefficient of friction of 0.1 was recorded for PTFE/PEEK composite coatings in comparison to coefficients of friction of 0.4 and 0.3 for pure PEEK and PEEK/graphite coatings. It also showed a three-times-lower wear rate than that of pure PEEK coatings. The hybrid nanocomposite (PEEK/PTFE/graphite) coatings were found to exhibit a slightly higher wear rate than the PEEK/PTFE coating due to the low bonding strength exhibited by the graphite sheet and PEEK matrix. For tests performed at a normal load of 10 N, the coefficient of friction decreased to less than 0.1 for the PEEK/PTFE composite coating when compared to the pure PEEK and hybrid nanocomposite coatings. However, the wear rate showed that the hybrid nanocomposite coating performed well, exhibiting the lowest wear rate due to the well-known load-carrying capacity of graphite and the exceptional lubricating capacity of the PTFE.

Cao et al. [63] studied the effect of tantalum nitride (TaN) nanoparticles on the tribological properties of PEEK coatings deposited by electrophoretic deposition. After drying, all the coatings were heat-treated at 390 °C for 60 min at a heating rate of 4.5 °C followed by cooling to ambient temperature. The friction and wear tests were performed against a ZrO_2_ counterface ball (Ø = 5 mm) using a reciprocating ball on disk tribometer. The tests were performed under a normal load of 5 N and a sliding speed of 5 Hz for 120 min. A 31.25% reduction in the COF (0.62 to 0.44) and an 82.80% reduction in the value of specific wear rate (94.2 × 10^−6^ to 16.2 × 10^−6^ mm^3^/Nm) was recorded for TaN/PEEK composite coatings (3 wt% TaN) in comparison to the pure PEEK coatings, thereby exhibiting excellent wear and lubrication effects provided by the TaN. The hardness values of the TaN/PEEK composite coatings (3 wt% TaN) were also found to be increased by 34% (0.35 GPa) compared to the pure PEEK (0.26 GPa).

Huang et al. [64] deposited (borated tantalum) TaB_2_/PEEK composite coating on pure titanium (TA2) substrate by the electrophoretic technique. After the coating, all the samples were heat treated at 390 °C for 60 min at a heating rate of 4.5 °C/min, followed by cooling to ambient temperature. The tribological testing was conducted on a reciprocating ball-on-disc configuration under lubricated conditions by using a simulated body fluid (SBF solution). The tests were conducted at a normal load of 5 N at a sliding speed of 5 Hz for 120 min using ZrO_2_ ball (Ø = 5 mm) as the counter face. The wear rate of TaB_2_/PEEK (3 wt% TaB_2_) coating in the simulated body fluid (SBF) was 72% lower than that of the PEEK coating, with a COF of 0.164 and a wear rate of 1.45 × 10^−6^ mm^3^.N^−1^.m^−1^, respectively. An improved hardness values were also observed by the addition of TaB_2_ when compared to pure PEEK coatings, with the 3 wt% reinforcement exhibiting the highest value. A summary of the tribological evaluations focused on the above section is provided in Table 10 and Table 11 based on the testing configuration at which the studies were conducted.

C. **Tribological Applications**

Lebga-Nebane et al. [16] deposited PEEK/h-BN composite and PEEK/hBN/tungsten carbide—cobalt chromium (WC-CoCr) hybrid composite coatings were prepared through hot-pressing onto steel substrates at 400 °C using a compression molding press. Two types of coatings were studied: single-layered and double-layered structures, with the latter involving the application of bottom and top-layer compositions sequentially. The tribological tests were conducted using a commercial tribometer equipped with a linear reciprocating ball-on-flat plane geometry. A steel ball (Ø = 6.3 mm) was used to slide back and forth over the test surface under a normal force of 1 kgf at room temperature without lubrication. Each test was performed at a speed of 5 cycles per second (300 rpm) with a stroke length of 10 mm in each direction for 36 min, corresponding to 10,800 cycles. The unpolished PEEK/h-BN double-layered coating exhibited the lowest coefficient of friction, measuring 0.21 when compared to the hybrid coatings developed in this study. Additionally, the same unpolished double-layered coating with the highest surface concentration of h-BN also showed a lower wear rate. Al_2_O_3_/PEEK composite coatings were applied by Moskalewicz et al. [50] using the electrophoretic deposition method to the Ti-6Al-4V substrate. The samples that had the coatings applied as-deposited underwent a 20 min heat treatment at 380 °C (4.5 °C/min). Two different cooling rates were used: one in room-temperature water and the other in a furnace with a cooling rate of 2 °C/min. A Vickers indenter was applied with a maximum load of 100 mN and a dwell time of 15 s to determine the hardness values of the coatings. A Rockwell C indenter with a 200 μm diamond tip radius was used to evaluate the coatings’ scratch resistance. The indenter was moved at a rate of 5 mm/min as the linear load increased from 0.03 to 30 N. The scratch length measured was 5 mm. Using a ball on disc tribometer, the friction and wear tests were conducted against Al_2_O_3_ balls. At room temperature, 150 °C, and 260 °C, with a sliding distance of 2000 m, the tests were conducted with a standard load of 5 N and a sliding velocity of 0.05 m/s. Coatings with a semi-crystalline, both Al_2_O_3_/PEEK (0.35 GPa) and pure PEEK (0.32 GPa) demonstrated higher hardness values than those with an amorphous structure (0.22 GPa for composite and 0.19 GPa for pure PEEK). Similarly, coatings with a semi-crystalline structure demonstrated a higher scratch resistance as well when compared to the coatings having an amorphous structure. At room temperature, the Al_2_O_3_/PEEK composite coatings showed a coefficient of friction of 0.25, whereas the pure semi-crystalline PEEK and pure amorphous PEEK coatings showed coefficients of friction of 0.3 and 0.33, respectively. Additionally, at room temperature, it demonstrated a five-fold reduction in wear rate compared to pure semi-crystalline PEEK coatings. The Al_2_O_3_/PEEK composite coating’s coefficient of friction rose to 0.37 and its wear rate increased seventeen times at 150 °C compared to room temperature. Tests conducted at 260 °C showed that the Al_2_O_3_/PEEK composite coating’s coefficient of friction rose to 0.35 and that its wear rate increased by 70 times compared to room temperature.

Hedayati et al. [51] studied the tribological properties of PEEK coatings reinforced with SiO_2_ nanoparticles deposited on plain carbon steel (st 37) using an electrostatic spray coating technique. The coated samples were transferred into an oven for post-heat treatment (30 min at 430 °C for pure peek and 50 min at 450 °C for nanocomposite coatings). Semi-crystalline coatings were obtained by cooling the coatings to room temperature, while amorphous coatings were obtained by quenching the as-melted coatings in an ice-water medium. Vickers microhardness was used to determine the hardness measurement at a load of 25 gf. The friction and wear tests were done on a pin-on-disc configuration at varying loads (3, 7, and 11 N) and a sliding speed of 0.13 m/s corresponding to a sliding distance of 1000 m. Hardness values were found to increase with the addition of SiO_2_. The semi-crystalline PEEK exhibited a higher hardness value (~19 HV) than the amorphous PEEK coatings (~15 HV). However, there were no comparable differences in the hardness values of the semi-crystalline nanocomposite coating (~26 HV) and the amorphous nanocomposite coating (~25 HV). The COF of the semi-crystalline and amorphous nanocomposite coatings was observed to be higher than the pure ones. Regardless of the crystalline structure of the matrix, after incorporating SiO_2_ particles, the wear resistance of the coatings improved. At all three loading conditions, the semi-crystalline nanocomposite coatings exhibited higher wear resistance than the amorphous nanocomposite coatings.

Zhang et al. [53] deposited SiC/PEEK composite coatings on an aluminum substrate using the printing technique. After depositing the powders, the coatings were heated to 370 °C for 5 min and then quenched into water at room temperature to obtain the desired coating having a thickness of 40 µm. Friction and wear tests were conducted on a ball-on-disc arrangement at varying loads (1, 5, and 9 N) and varying sliding velocities (0.2, 0.5, 0.8, 1.1, and 1.4 m/s), corresponding to a total sliding distance of 2000 m. Under low load (1 N), the friction coefficients decrease slightly while increasing the sliding velocity for pure PEEK coatings whereas, under high load (5 and 9 N) and low velocities, the COF remains almost the same. The SiC/PEEK composite coatings exhibited a slightly higher COF than pure PEEK coatings at lower loads but as the loading increased, it was found to cause a decrease in the values of COF. A lower wear rate was evident with the introduction of SiC nanoparticles. As the sliding velocities increased, the nanocomposite coatings exhibited higher wear resistance compared to pure PEEK coatings.

Tharajak et al. [55] studied the friction/wear properties of PEEK coatings reinforced with hexagonal boron nitride (h-BN) deposited over low carbon steel (AISI 1040) with the flame-spray technique. A Vickers microhardness test setup (load = 300 gf, dwell time = 15 s) was used to determine the coating’s hardness values. The effect of different particle sizes (0.1, 0.5, and 1.5 µm) on the hardness values of the coatings were also investigated. Friction and wear tests were carried out using a ball-on-disc configuration for varying normal loads (5 & 25 N) and a sliding speed of 0.1 m/s for a sliding distance of 1000 m. The addition of h-BN with a particle size of 0.5 μm provided the highest hardness value. The specific wear rate was lowered by the addition of h-BN particles at an applied load of 5 N. Furthermore, the friction coefficient was lowered by adding larger amounts of h-BN, particularly 8 wt% h-BN to the PEEK covering. Since h-BN had a strong solid lubricating capacity, 8 wt% h-BN/PEEK composite coatings exhibited good efficiency of friction reduction for an applied load of 25 N. In addition, h-BN/PEEK coating with h-BN particle size of 0.1 μm exhibited the lowest specific wear rate, whereas h-BN/PEEK composite coatings filled with larger-size h-BN particles (0.5 and 1.5 μm) revealed typical sliding grooves from the abrasive wear mechanism.

Moskalewicz et al. [57] deposited titanium nitride (TiN)/PEEK composite coatings on the Ti-6Al-4V substrate by the electrophoretic deposition technique. Samples with the as-deposited coatings were heat treated at a temperature of 390 °C for 40 min (heating rate of the samples 4.5 °C/min) and were cooled down in a furnace (cooling rate of 2 °C/min) after soaking. The scratch resistance of the coatings was tested using a Rockwell C indenter with a diamond tip radius of 200 μm for a scratch length of 5 mm and an indenter displacement rate of 5 mm/min for an increasing linear load from 0.03 to 30 N. The friction and wear tests were done on a ball-on-disc configuration for a normal load of 5 N at a rotational speed of 140 RPM corresponding to a sliding distance of 2000 m. No cohesive cracks or adhesive failures were observed in the composite coatings up to the maximum load of 30 N, indicating excellent scratch resistance. The TiN/PEEK708 coatings reduced the coefficient of friction from 0.70 (baseline alloy) to 0.30, indicating a significant improvement in frictional behavior. The wear rate of the TiN/PEEK-coated oxygen hardened alloy was approximately 70 and 650 times lower compared to the oxygen hardened and baseline alloy respectively.

Li et al. [61] deposited CF/PEEK composite coatings over 17-4PH martensitic stainless steel by the flame-spray technique. Two types of short carbon fibers (CFs) were used: 300-mesh CFs with intact morphology and 600-mesh CFs with fractured shape and higher graphitization degree. The tribological studies were conducted on a ball-on-disc configuration under normal loads of 5 and 7 N at sliding velocities of 0.2 m/s and 0.5 m/s over a sliding time of 120 min. 300-mesh CFs increased surface hardness by 1.31 times compared to pure PEEK coatings, while 600-mesh CFs led to significantly lower specific wear rates due to higher graphitization degree. The severe wear of the flame-sprayed CF/PEEK composite coatings with carbon fibers of 300 and 600 mesh in 30 wt% content is observed under the different loads and sliding speeds. The average friction coefficient of CF/PEEK composite coatings with different CF types and contents slightly increases with higher loads. Coatings with 10 wt% CF content also showed optimized nanoindentation behavior and tribological properties.

Zhu et al. [69] deposited PEEK/PTFE composite coating over a stainless-steel substrate by electrostatic powder spray technique. After spraying the powders, all the coated samples were transferred into an oven for post-heat treatment at 380 °C for 5 min. The coatings were then quenched into ice water medium and annealed for 30 min at 260 °C in an oven to obtain a uniform coating with a thickness of 200 µm. Friction and wear tests were performed on a ball-on-disc arrangement with a 100Cr6 steel ball (Ø = 6 mm) as the counter face. The tests were conducted at a normal load of 10 N, corresponding to a sliding distance of 697 m. The PTFE/PTFE composite coating (3 wt% PTFE) exhibited a COF of 0.133 in comparison to the COF of 0.149 for pure PEEK coatings. An improved wear resistance was observed by the addition of PTFE (38 × 10^−6^ mm^3^/Nm) when compared to pure PEEK coatings (44 × 10^−6^ mm^3^/Nm). However, the addition of PTFE was found to have no improvement in the hardness values.

Kadiyala et al. [73] studied the influence of nano and micron sized SiC particles on PEEK coatings deposited over 316 stainless steel using an electrostatic powder spray technique. After coating, the samples were transferred into an oven at 370 °C for 30 min followed by cooling it to room temperature. Scratch tests were conducted at constant load test conditions (2 to 8 N, slider speed = 0.33 mm/s and traversing time = 15 s) and dynamic loading conditions (slider speed = 0.33 mm/s and loading rate = 2 N/min). The scratch hardness and adhesion of coatings improved significantly by two times when compared to pure PEEK coatings with an increase in micro-particles of SiC contents and the optimum concentration was found to be around 10–15 wt%. The nanoparticles (3 wt%) in composite coatings showed significantly (two-times) improved friction and wear behavior compared to their microparticles (3 wt%) counterparts. The inclusion of SiC particles also significantly improved the load bearing capacity of the coatings.

Yeo and Polycarpou et al. [83] studied the tribological performances of PTFE/PEEK composite coatings under oilless compressor conditions deposited over gray cast iron using an electrostatic powder spray technique. The friction and wear tests were conducted on a pin-on-disc configuration at a normal load of 445 N under oscillatory (load = 445 N, linear velocity = 0.22 m/s) and unidirectional motion (load = 445 N, linear speed = 3.75 m/s). Better performance with lower COF values and wear rates was obtained under unidirectional conditions than oscillatory operating conditions.

Nunez et al. [84] deposited PEEK/PTFE composite and PEEK/PTFE/Ceramic blend hybrid composite coatings over gray cast iron substrate by electrostatic spray gun technique. Friction and wear tests were performed by using a high-pressure tribometer with a pin-on-disc configuration under lubricated conditions. Experiments were conducted at a normal load of 100 N for a test duration of 60 min under a sliding velocity of 4.8 m/s. A low values of COF of 0.06 and 0.07 were obtained for PEEK/PTFE/ceramic blend hybrid and PEEK/PTFE composite coatings. The wear tests concluded that the PEEK/PTFE composite coatings exhibited only mild burnishing whereas the PEEK/PTFE/ceramic blend hybrid coatings developed wear depths of ~10 μm corresponding to the wear scars reporting depths of ~40 μm. A summary of the tribological evaluations focused on the above section is provided in Table 12.

## 3. General Conclusions and Recommendations

Polyether ether ketone (PEEK) is one of the high-performance polymers that has gained significant attention in recent years due to its exceptional mechanical properties, chemical resistance, and thermal stability, making it a prominent candidate for applications in industries. However, PEEK, in its pristine form, exhibits poor wear resistance with a moderate coefficient of friction (0.30–0.38). Many attempts have been made by several researchers to improve its wear resistance and lower the COF by developing composite coatings. Hence, the current review not only summarizes those efforts but also present a detailed tribological evaluation of PEEK pristine and PEEK composite coatings in terms of the various approaches adopted by the researchers to improve the properties of PEEK, the different types of reinforcements and various dispersion techniques used to develop PEEK composite coatings. By consolidating and analyzing the existing knowledge, the following insights are offered that would be helpful in the development of more durable, high-performance PEEK nanocomposite coatings for a broad range of tribological applications.

- The choice of filler/reinforcing material(s) depends on targeted application. However, the concentration of filler material must be optimized to obtain desired coating characteristics.
- It is observed that uniform dispersion of reinforcement/fillers has a profound effect not only on coating morphology but also on its overall properties. Wet mixing, which involves the usage of high-frequency ultrasonic waves, was observed to promote uniform dispersion of fillers/reinforcement in PEEK matrix.
- Among the various deposition techniques, electrophoretic deposition was observed to be most popular for depositing PEEK composite coatings intended for bio-medical applications due to the non-uniform shape and the limited size of the implant. On the other hand, electrostatic spray coating and flame-spray coatings are preferred to deposit PEEK coatings intended for industrial tribological applications owing to their simplicity and cost-effectiveness. However, deposition/spraying parameters must be optimized to obtain the desired coating characteristics.
- It was noted that mere deposition results in poor mechanical properties due to the formation of highly porous films with weak adhesion to the substrate. Post-deposition treatment processes, such as laser re-melting and heat treatment in an oven, emerged as effective in eliminating/diminishing pores, rendering dense, homogeneous, and continuous coatings. However, process parameters must be optimized to obtain desired coating characteristics.
- The cooling rate affects the degree of crystallinity of coating which may have different effects on mechanical and tribological properties. Slow cooling rates after post-deposition heat treatment results in semi-crystalline structures, which has a positive effect on mechanical and tribological properties of coatings in comparison the amorphous structure resulting from faster cooling rates.
- PEEK based composite/hybrid coatings on metallic substrates reported in literature can be classified as coatings for bio-structural, bio-tribological, and tribological applications. The coatings designed for bio-structural applications (structural component of implant) focus on enhancement of bioactivity, antibacterial properties, corrosion resistance, and adhesive strength. The coatings designed for bio-tribological applications where the implant is expected to experience relative motion in service contain hard particles which are biocompatible. They focus on enhancement of friction and wear properties along with electro-mechanical properties. The coatings designed for tribological applications in industry focus on improvement in corrosion, friction, and wear properties.
- Though extensive research has been conducted in developing PEEK based composite coatings for bio-structural applications, very few studies attempted to evaluate properties relevant to their tribological performance.
- Very few researchers have developed PEEK based composite/hybrid coatings for industrial tribological applications leaving a wide gap in literature for further exploration.
- PEEK-based hybrid coatings hardly received any attention for any reported application. Therefore, it is recommended to develop the same and evaluate its tribological performance in future studies.

## Figures and Tables

**Table 1:** Structural, mechanical and thermal properties of PEEK [21,22].

Property	Value
Structure	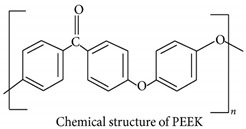
**Mechanical**	
Elongation at Break (%)	20–40
Impact Strength (KJ/m^2^)	5–8.5 at 20 °C
Modulus of Elasticity (GPa)	3.3–4.0
Shear Stress (MPa)	90–120
Coefficient of friction	0.3–0.38
Density (g/cm^3^)	1.3 ± 0.02
Elongation (% strain)	0.3–1.5
**Thermal**	
Coefficient of Thermal Expansion	4.7–6 × 10^−6^ K^−1^
Melting Point (°C)	340–343
Specific Heat Capacity (J/(Kg K))	2100–2200 at 20 °C
Maximum Working Temperature (°C)	239–260
Poisson’s Ratio	0.43

**Table 2:** Some of the potential industrial applications of PEEK.

Industry/Applications	Key Utility
Oil and Gas	Chemical, heat resistance
Fiber Optics	Strength and temperature resistance
Fluid Management	Chemical resistance
General Industries	Toughness, high melting point
Replacement for metals	Weight factor

**Table 3:** Overview of major PEEK manufacturers, product lines, and key applications (data acquired from corresponding websites).

Manufacturer	Country of Origin	Product Lines	Key Applications
Victrex	UK	VICTREX PEEK, PEEK-based composites	Aerospace, Automotive, Electronics, Oil and Gas, Medical
Solvay	Belgium	KetaSpire PEEK	Aerospace, Healthcare, Industrial, Consumer Goods
Evonik	Germany	VESTAKEEP PEEK	Medical, Automotive, Aerospace, Electronics
LyondellBasell	Netherlands	PEEK and high-performance polymer compounds	Automotive, Electronics, Industrial Applications

**Table 4:** Effect of post-deposition treatment on coating characteristics.

Deposition Technique	Substrate	Coating Characteristics	Post-Deposition Treatment	Coating Characteristics After Post-Deposition Treatment	Ref.
Flame-Spray	SS	Large porosity, thin film, weak adhesion, poor mechanical properties	Nd:YAG laser remelting	Dense and homogeneous coating	[30]
			CO_2_ Laser remelting	No complete elimination of porosities near interface	
	Al	High porosity, poor mechanical properties	Nd:YAG laser remelting	No complete elimination of porosities near interface	[1]
			CO_2_ Laser remelting		
	SS		Nd:YAG laser remelting	Diminished porosities near interface	
			CO_2_ Laser remelting	Dense and homogeneous coating	
Electrophoretic	Ti-6Al-4V	Not available	Heat treatment in oven	Dense, homogeneous and continuous	[37]

SS: Stainless Steel; Al: Aluminium.

**Table 5:** Classification and characteristics of reinforcements used in PEEK composite/hybrid coatings.

Material	Reinforcement	Characteristics	References
Bioactive	Hydroxy Appetite	Bioactive agent, bone bonding capability, accelerates bone cell growth	[39,40]
	Bioglass	Bioactive agent, rapid bonding with bone, accelerates bone cell growth	[33,41,42,43,44,45]
		Degradable in body	
Ceramic	Al_2_O_3_	Hard, chemically inert, corrosion resistant, cytocompatible	[46,47,48,49,50]
	SiO_2_	Hard, chemically inert, biocompatible	[51]
	k-SiO_2_	Hard, chemically inert, hydrophobic	[52]
	SiC	Hard, chemically inert	[53]
	Si_3_N_4_	Hard, bioactive, accelerates bone repair, antibacterial effect	[54]
	h-BN	Self-lubricating, inert, high thermal conductivity, high thermal stability	[45,55,56]
	TiN	Hard, inert, stable, low friction coefficient	[57]
	WC-CoCr	Hard, chemically inert	[16]
Carbon Based	Graphite	Good load bearing capability, solid lubricant,	[15,58]
		high electrical and thermal conductivity	
	GO	High strength and elastic modulus, excellent thermal and electrical conductivity	[17,59]
	RGO	High strength and elastic modulus, excellent thermal and electrical conductivity	[60]
	Carbon Fibre	High stiffness, tensile strength, strength–weight ratio,	[17,61]
		thermal and chemical stability	
Refractory	Ta	High heat, wear and corrosion resistance, biocompatible, hemocompatible	[62]
	TaN	High heat, wear and corrosion resistance, biocompatible, hemocompatible	[63]
		Anti-bacterial effect	
	TaB_2_	High heat, wear and corrosion resistance, anti-inflammatory effect	[64]
	Ag	High thermal and electrical conductivity, anti-bacterial, and anti-microbial effects	[44]
Metallic	ZnS	High stability, non-toxic, anti-bacterial, and anti-microbial effect	[39]
	MoS_2_	Self-lubricating, anti-bacterial effect against certain pathogens	[40]
Polymer	PTFE	Self-lubricating, hydrophobic, chemically inert	[52,65,66]
Minerals	Huntite	Flame retardant material with high decomposition temperature	[66]

Al_2_O_3_: alumina; SiO_2_: silicon dioxide; SiC: silicon carbide; Si_3_N_4_: silicon nitride; h-BN: hexagonal boron nitride; TiN: titanium nitride; WC: tungsten carbide; GO: graphene oxide; RGO: reduced graphene oxide; Ta: tantalum; TaN: tantalum nitride; TaB_2_: tantalum diboride; ZnS: zinc sulfide; MoS_2_: molybdenum disulfide.

**Table 6:** Dispersion parameters used for wet mixing of powders.

Solvent	Particles to Disperse	Dispersing Time	Mixing Time	Solvent Evaporation	Coating Technique	Ref.
Ethanol	Ta + PEEK	30 min in ultrasonic bath	30 min magnetic stirring	Not Applicable	Electrophoretic	[62]
	Al_2_O_3_ + PEEK	30 min in ultrasonic bath	7 min magnetic stirring			[46]
	TaN + PEEK	30 min in ultrasonic bath	30 min magnetic stirring			[63]
	Bioglass + PEEK	30 min in ultrasonic bath	5 min magnetic stirring			[41]
	PTFE + PEEK	15 min in ultrasonic bath	10 min magnetic stirring			[66]
	ZnS + HA + PEEK	20 min in ultrasonic bath	10 min magnetic stirring			[39] *
	Graphite + PEEK	15 min in ultrasonic bath	5 min magnetic stirring			[58]
	Al_2_O_3_ + PEEK	20 min in ultrasonic bath	5 min magnetic stirring			[49]
	Si_3_N_4_ + PEEK	20 min in ultrasonic bath	10 min magnetic stirring			[54]
Ethanol + isopropanol	GO + PEEK	6 min probe-sonication	24 h magnetic stirring			[59]
	RGO + PEEK	6 min probe-sonication	24 h magnetic stirring			[60]
Ethanol	MoS_2_ + PEEK	20 min in ultrasonic bath				[74]
	MoS_2_ + PEEK + HA	10 min in ultrasonic bath	10 min magnetic stirring			[40] *
Ethanol	Al_2_O_3_ + PEEK	20 min in ultrasonic bath	5 min magnetic stirring			[50]
Ethanol	Bioglass + Ag + PEEK	3–5 min in ultrasonic bath	3 min magnetic stirring			[44] *
	Bioglass + PEEK	1 h in ultrasonic bath	5 min magnetic stirring			[33] *
	Bioglass + h-BN + PEEK	30 min in ultrasonic bath	5 min magnetic stirring			[45] *
Ethanol	SiC	30 min probe-sonication			Electrostatic Spray	[73]
	SiC + PEEK	30 min probe-sonication		Dried in Oven		
Ethanol	PTFE + PEEK		10 min magnetic stirring			[52]
	PTFE + PEEK + k-SiO_2_		15 min magnetic stirring	80 °C for 12 h		
Ethanol	CF + PEEK	2 h in ultrasonic bath		60 °C for 6 h	Flame-Spray	[61]
Ethanol	CNT + PEEK	2 h in ultrasonic bath		60 °C for 6 h		[20]

*: Magnetic stirring performed first followed by sonication.

**Table 7:** Processing parameters used in electrophoretic deposition of PEEK composite/hybrid coatings.

Filler	Substrate	Trial Deposition		Optimal Deposition		Post-Deposition Heat Treatment	Coating Thickness	Ref.
		Voltage	Time	Voltage	Time			
Ta	Ti			20 V	180 s	390 °C for 60 min followed by furnace cooling to room temperature		[62]
Al_2_O_3_	316 SS	20, 30, 40 V	180 s	30 V	180 s	343 °C at the heating rate of 10 °C/min for a holding time of 30 min		[46]
TiO_2_	316 SS	8–56 V	1–10 min	30 V	60 s	335 °C at the heating rate of 10 °C/min for a holding time of 30 min	8–10 µm	[72]
TaN	Ti	15–25 V	30–180 S	20 V	180 s	390 °C at the heating rate of 4.5 °C/min for a holding time of 60 min		[63]
PTFE	Ti Alloy	10–100 V	20–100 s	90 V	40 s	450 °C for a holding time of 20 min followed by cooling: (a) at rate of 2 °C/min, (b) immediately in water to room temperature	45 µm	[66]
Bioglass	NiTi wires			20 V	300 s	340 °C at the heating rate of 5 °C/min for a holding time of 20 min	15 µm	[41]
ZnS + HA	Zr Alloy	10–150 V	30 s	90 V	30 S	450 °C at the heating rate of 15 °C/min for a holding time of 30 min followed by furnace cooling at the rate of 2 °C/min	55–60 µm	[39]
Huntite + Al_2_O_3_	304SS			50 V		340 °C for a holding time of 60 min		[48]
Graphite	Ti Alloy	10–100 V	40 s	70 V	40 s	390 °C at the heating rate of 4.5 °C/min for a holding time of 40 min followed by furnace cooling at the rate of 2 °C/min		[58]
Bioglass	Ti Alloy			50–65 V	120 s	355 °C at the heating rate of 10 °C/min for a holding time of 60 min followed by furnace cooling	40 µm	[42]
Al_2_O_3_	Ti Alloy	30–70 V	10–60 s	50 V	20 s	350 °C at the heating rate of 4.5 °C/min for a holding time of 20 min followed by furnace cooling at the rate of 2.7 °C/min	45–120 µm	[49]
Si_3_N_4_	Ti Alloy	10–20 V	30–120 s	15 V	90 s	355 °C at the heating rate of 4.5 °C/min for a holding time of 30 min followed by furnace cooling at the rate of 2 °C/min	120 µm	[54]
GO	SS	10, 30 V	1–5 min	30 V	180 s	380 °C at the heating rate of 10 °C/min for a holding time of 5 min followed by furnace cooling at the rate of 2 °C/min		[59]
RGO	SS316			30 V	180 s	380 °C at the heating rate of 10 °C/min for a holding time of 5 min followed by furnace cooling at the rate of 2 °C/min		[60]
TiN	Ti Alloy	30–100 V	30–90 s	90 V	30 s	390 °C at the heating rate of 4.5 °C/min for a holding time of 40 min followed by furnace cooling at the rate of 2 °C/min	120 µm	[57]
HA + MoS_2_	Ti Alloy	50–150 V	30 s	90 V	30 s	390 °C at the heating rate of 4.5 °C/min for a holding time of 40 min followed by furnace cooling at the rate of 2 °C/min	30–35 µm	[40]
Al_2_O_3_	Ti Alloy	20–100 V	40 s	70 V	40 s	380 °C for a holding time of 20 min followed by cooling: (a) at a rate of 2 °C/min, (b) Immediately in water to room temperature	(a) 95 µm	[50]
							(b) 80 µm	
Bioglass + Ag	SS			200 V/cm	120 s	380 °C for a holding time of 60 min		[44]
Bioglass	316SS			110 V	120 s	400 °C at the heating rate of 2 °C/min for a holding time of 30 min followed by furnace cooling at the rate of 2 °C/min	80 µm	[33]
Bioglass + h-BN	316SS			90 V	60 s	375 °C at the heating rate of 2 °C/min for a holding time of 30 min		[45]

**Table 8:** Spraying parameters of neat and composite PEEK coatings applied by an electrostatic spray-coating process.

Filler	Substrate	Applied Voltage (KV)	Applied Current (µA)	Nozzle-to-Substrate Distance (mm)	Air Pressure (MPa)	Powder Feed Rate (g/min)	Post-Deposition Heat Treatment	Thickness (µm)	Ref.
GO + CF	Ti Alloy	60	180	50	0.01	20	385 °C at the heating rate of 5 °C/min for a holding time of 1 h followed by natural cooling to room temperature	50–150	[17]
SiO_2_	CS	70		250	0.2	16	430 °C for 30 min for pure PEEK	150	[51]
							450 °C for 50 min for composite		
PTFE	SS	90	85	200	0.4		380 °C for 5 min followed by quenching in ice water with subsequent annealing at 260 °C for 30 min	200	[69]
SiC	SS316	90			0.175		370 °C for 30 min followed by natural cooling to room temperature	150	[73]

**Table 9:** Spraying parameters of neat and composite PEEK coatings applied by a flame-spray coating process.

	Parameters								Coating Thickness (µm)	Ref.
Filler	Substrate		Gas			Spray				
	Material	Preheat	Gas	Pressure (MPa)	Flow Rate (L/min)	Distance (mm)	Gun Speed (mm/s)	Time (s)		
h-BN	AISI 1040	200 °C	C_3_H_8_	0.45	27.7	120	80	20	250–300	[55]
			O_2_	0.41	47.34					
			N_2_	1.1						
CF	SS	200 °C	C_2_H_2_	0.14	2	200	300		150–200	[61]
			O_2_	0.7	1					
			Air	0.4						
CNT	SS	200 °C	C_2_H_2_	0.14	2	200	300		150–200	[20]
			O_2_	0.7	1					
			Air	0.4						

**Table 10:** Tribological evaluations of the coatings performed under linear sliding conditions.

Filler	Substrate	Load (N)	Speed (m/s)	Distance (m)	COF	Sp. Wear Rate (×10^−6^ mm^3^/Nm)	Ref.
Bioactive Glass	316 L Stainless Steel	7	NA	NA	0.37	NA	[33]
Alumina	Titanium Alloy	5	0.075	1000	0.29	1.9	[49]
PTFE	Titanium Alloy	10	0.05	180	0.08	6.3	[15]
Graphite					0.31	7.75	

PTFE—Polytetrafluoroethylene.

**Table 11:** Tribological evaluations of the coatings performed under reciprocating conditions.

Filler	Substrate	Load (N)	Speed (Hz)	Time (min)	COF	Sp. Wear Rate (×10^−6^ mm^3^/Nm)	Ref.
GO/CF	Titanium alloy	5	2	60	0.08	NA	[17]
TaN	Titanium alloy	5	5	120	0.44	1.62	[63]
TaB_2_	Titanium alloy	5	5	120	0.164	1.45	[64]

TaN—tantalum nitride, TaB_2_—borated tantalum, GO—graphene oxide, CF—carbon fiber.

**Table 12:** Tribological evaluations of the coatings performed under linear sliding conditions.

Filler	Substrate	Load (N)	Speed (m/s)	Distance (m)	COF	Sp. Wear Rate (×10^−6^ mm^3^/Nm)	Ref.
Al_2_O_3_	Titanium alloy	5	0.05	2000	0.25	0.47	[50]
SiO_2_	Carbon Steel (st 37)	3	0.13	1000	0.4	30	[51]
h-BN	Low Carbon Steel	5	0.1	1000	0.2	14	[55]
		25			0.22	37	
TiN	Titanium alloy	5	0.04	2000	0.3	1.1	[57]
PTFE	Stainless Steel	10	NA	697	0.1328	38.57	[69]
SiC	Aluminum disc	9	1.4	2000	0.27	25	[53]
PTFE	Grey Cast Iron	100	4.8	8640	0.07	0.9	[84]
PTFE/Ceramic Blend					0.06	3.8	

PTFE—polytetrafluoroethylene, SiO_2_—silicon dioxide, SiC—silicon carbide, TiN—titanium nitride, Al_2_O_3_—alumina.
